# The Future is Local: the Role of Decentralised Clinical Trials in Patients with Advanced Cancer Receiving Palliative Care

**DOI:** 10.1007/s11864-026-01404-1

**Published:** 2026-07-27

**Authors:** Claire Stokes, Phillip Good

**Affiliations:** 1 Mater Hospital Brisbane, Level 6 Duncombe Building, Raymond Terrace, South Brisbane, 4101 Australia; 2 Mater Research Institute, University of Queensland, Brisbane, Australia; 3 St Vincent’s Private Hospital Brisbane, Kangaroo Point, Australia; 4 Centre for Palliative Care Research and Education, RBWH, Herston, Australia

**Keywords:** Decentralised clinical trial, Teletrial, Advanced cancer, Clinical research, Palliative care

## Abstract

Trial participation is increasingly recognised as a crucial component of optimal cancer care, yet patients with advanced cancer receiving palliative care often face significant challenges in meeting the logistical demands of traditional clinical trials. Decentralised clinical trials (DCTs), including teletrials, which conduct study activities outside a central site – frequently in participant’s homes or local communities - offer a promising model to overcome these barriers. By enabling remote participation, DCTs can overcome geographical and functional barriers, making trials more accessible to this often-frail population. However, there are important concerns that warrant careful consideration. DCTs may inadvertently fragment care, expose vulnerable patients to novel medications in an isolated setting, and exacerbate digital health inequities. In our experience these challenges can be mitigated through careful trial selection, as well as the implementation of rigorous screening and support systems to ensure both patient suitability and continuity of care. Key measures include close liaison with treating oncologists and palliative care teams, 24/7 clinical phone support, accessible IT assistance during working hours, and collaboration with local trial coordinators when available. This approach not only extends the geographical reach of clinical trial participation for patients with advanced cancer receiving palliative care, but also maintains trial rigour and patient safety.

## Introduction

Participation in clinical trials is increasingly recognised as an integral component of optimal cancer care, providing access to innovative therapies prior to regulatory approval [1–3]. Additional benefits may result from enhanced access to specialised nursing and medical care, treatment at high-volume centres, and improvements in routine care delivered within a trial setting [4–10]. While the benefits of clinical trial participation are well established in medical oncology, the appropriateness and acceptability of clinical trials in patients with advanced cancer receiving palliative care have been questioned [11–14]. This population represents a vulnerable cohort who may struggle to adhere to the logistical and temporal demands of clinical research [13, 14]. These challenges may be amplified for patients living in regional, rural, and remote areas [15–18]. Yet access to clinical trials for patients with advanced cancer receiving palliative care is associated with improved quality of life and decreased symptom burden [19]. This highlights the need for clinical trial models that mitigate the functional and geographic barriers experienced by this population. Decentralised clinical trials (DCTs), including teletrials, represent a promising strategy to address these challenges. This article discusses the feasibility, facilitators, barriers, advantages, disadvantages and ethical considerations surrounding DCTs, with a view to considering their role in patients with advanced cancer receiving palliative care.

### Decentralised Clinical Trials

Decentralised clinical trials (DCTs) are a model of trial delivery in which some or all trial-related activities occur away from the centralised trial site [20]. By leveraging technology and other tools, DCTs reduce or eliminate the need for in-person interaction between participants and central site researchers, shifting much of the activity to the patient’s home or local community [21–23].

Technological and societal advances in the late 1990s paved the way for the first internet-based DCTs in the early 2000s [24–26]. More recently, greater availability of mobile devices and the COVID-19 pandemic have fuelled an increase in the number of DCTs being conducted, with a 2021 systematic review including 45 decentralised randomised controlled trials (RCTs), of which 28 (62%) were fully decentralised [21, 27, 28]. Decentralised processes, such as digital consent, randomisation, and remote data collection are increasingly used to complement conventional trial design [22].

Teletrials, developed by the Clinical Oncology Society of Australia, are a type of DCT in which smaller research centres, often located in regional, rural, and remote areas, are connected as satellite sites to a primary research site via telehealth [3]. These satellite sites become the focus of trial activity, with patients recruited and managed at their local site by local research staff, with primary site staff connecting via telehealth as required [23, 29, 30]. The principal investigator from the primary site assumes overall responsibility for trial activities across the sites [3, 22, 23, 29]. Teletrials broaden the type of trial that can adopt a decentralised approach, with those requiring some in-person clinical contact able to participate [23].

### Evaluation of Decentralised Clinical Trials

A 2021 systematic review of 45 DCTs concluded that DCTs are feasible, with a recruitment and retention rate averaging 141 (range 0 to 11,035) and 93% (range 1.1 to 100%) respectively [21]. Although this was not compared to conventional trials, analysis of early DCTs in non-cancer populations found that recruitment, retention and adherence rates were similar between the two groups [24, 25]. Subsequent DCTs have demonstrated rapid recruitment, often driven by social media campaigns with retention, compliance, and satisfaction rates typically exceeding 90% [31–33]. Teletrials have also demonstrated success in patient recruitment [34, 35]. Trials in patients with advanced cancer receiving palliative care tend to experience lower recruitment and retention rates due to the frailty of this patient cohort [14]. It is not known if DCTs outperform conventional trial models in this population.

Results from early post-participation surveys indicate enthusiasm for DCTs, with a majority of participants rating the trial process as good or excellent, and reporting that they would be very or extremely likely to participate in similarly designed trials in the future [24, 26, 36]. In a mixed-method analysis, rural and remote patients with cancer who participated in a teletrial reported no financial, social, or emotional burden, in contrast with those who travelled to a metropolitan centre who cited the travel as a significant negative factor [37].

Advocates of DCTs cite cost savings as a significant advantage of this model, however there is limited evidence to support this claim [21]. A systematic review of 45 decentralised RCTs calculated the cost per randomised patient as US$914, with a range from US$155 to US$3,400. These costs were not compared to those of conventional trials [21]. However, an early DCT estimated its costs to be less than half of those of similar conventional trials (US$914 per patient versus US$1,925 per patient), with savings in areas such as salaries, maintenance of physical premises, and travel reimbursement [24, 25].

### Facilitators and Barriers

Technological advances including the internet, increased access to mobile devices, and artificial intelligence have facilitated an increase in DCTs [21]. Conversely, poorly developed digital infrastructure, inadequate devices, lack of digital literacy, and technological malfunctions act as barriers [26, 38]. It is not known if patients with advanced cancer receiving palliative care, have decreased digital literacy when compared to the standard population.

Regulation, legislation and guidelines governing DCTs influence their feasibility in patients with advanced cancer receiving palliative care. Supportive and flexible frameworks that recognise the importance of minimising patient, caregiver and clinician/researcher burden facilitate participation, while difficult to interpret, costly to implement, inconsistent, or absent frameworks act as a barrier [21]. Similarly, logistical support, established palliative care research networks, home-based care infrastructure, digital connectivity, and the ability to integrate research tasks into routine palliative services act as important facilitators, while their absence creates barriers [21].

Societal attitudes towards remote research plays a key role; positive attitudes are a facilitator, while hesitancy, negative perceptions, limited experience, technological discomfort, and privacy concerns act as barriers [21]. Confusion around the role of the research versus the treating physician with possible fragmentation of care, and the perception of less person-oriented healthcare may be perceived unfavourably and contribute to DCT failure [38].

### Advantages and Disadvantages

DCTs can reduce physical and emotional burden for vulnerable participants by minimising travel, decreasing inpatient visits, and enabling participation for those who are homebound or have limited mobility [21, 22]. Features such as passive data collection, automation, and continuous wearable monitoring devices may enhance both convenience and safety [21, 22]. By broadening eligibility, DCTs can also improve the representativeness of trial populations [39].

However, DCTs are not without disadvantages in this population. Reduced in-person contact may weaken the doctor-patient relationship, leading to emotional isolation and diminished support [21, 22]. Data security concerns and reduced clinical oversight may compromise safety, while reliance on patient-collected data raises questions about validity and reliability [21, 22, 40]. Participants may face increased burden from tasks traditionally handled by research staff, such as data entry and device maintenance, and research centres may experience added pressure from new training and technological demands [21, 22]. Digital unfamiliarity, limited device access, cognitive decline, fatigue, and symptom burden may hinder participation [21, 22].

Teletrials can expand access to specialist care and research for regional and remote sites, but they introduce financial and operational challenges [3, 41]. Costs associated with medication transport, site visits, and establishing technological and pharmacy infrastructure can be substantial [3]. A lack of workforce stability, communication challenges, and difficulties accessing source documents between sites may affect trial quality. Safety concerns may arise when novel treatments are delivered in settings with limited clinical trial expertise [3].

### Ethical Considerations

DCTs in patients with advanced cancer receiving palliative care must uphold the bioethical principle of non-maleficence by ensuring safe handling of investigational products, biological specimens, and adverse-event monitoring, supported by appropriate risk-mitigation procedures [22, 42]. Conducting trials in the home raises concerns, as these environments are not assessed for clinical suitability [22]. Cyber security risks require stringent data-minimisation and protection, particularly when data is stored on personal devices [22, 42].

Electronic consent provides accessibility benefits and promotes equity, but poses challenges in verifying identity and ensuring that consent is truly informed for patients who may experience fatigue, cognitive decline, or limited digital familiarity [22, 42]. While many countries including the UK, Belgium, the USA and Australia have issued guidelines regarding the legality of electronic consent, the electronic process can be complex for some participants, which may hamper their ability to engage in the trial, thereby introducing digital inequity [22, 42, 43]. Multi-factor authentication and complex digital processes can add burden, compounding the shift of trial tasks from staff to participants and their caregivers [22, 26]. DCTs must carefully evaluate the direct and indirect costs of participation to avoid exploiting participant’s time and resources [42]. Trial results should always be communicated back to participants [22].

Teletrials can increase equity by supporting access to specialist care for regional or home-based palliative-care patients, but require careful site selection, clear delineation of workforce roles, appropriate training, and adherence to financial and regulatory requirements to ensure safe and reliable delivery of trial interventions [3].

DCTs should always address critical evidence gaps and be the most appropriate trial design for the research question [42]. Any decision to transition from a conventional trial to a DCT must be carefully justified based on the study’s characteristics, while weighing the benefits of improved patient access against concerns about participant safety and data quality [22]. Potential participants who cannot engage due to lack of digital literacy or infrastructure should be offered technology, training, and support, or be provided with an alternative, if they meet the inclusion criteria [22]. Increased ethical oversight may be necessary to identify and manage the novel risks associated with DCTs [42].

## Conclusion

Patients with advanced cancer receiving palliative care represent a particularly vulnerable population. Historically, clinicians have been hesitant to refer these patients for participation in clinical trials. Decentralised clinical trials, including teletrials, have the potential to reduce trial-related burdens and so increase clinical trial access. However, implementing such models in this population will require careful planning to ensure participant safety and adequate support.

## Key References

- Vayena EP, Blasimme AP, Sugarman JMD. Decentralised clinical trials: ethical opportunities and challenges. *Lancet Digit Health*. 2023;5:e390-e4.
  - ○ This reference is of great importance as it provides a comprehensive discussion of the ethical opportunities and challenges posed by DCTs. Such considerations are especially relevant for the vulnerable population of patients with advanced cancer receiving palliative care.
- Rogers A, De Paoli G, Subbarayan S, et al. A systematic review of methods used to conduct decentralised clinical trials. *Br J Clin Pharmacol*. 2022;88:2843-62.
  - ○ This reference is of great importance as it represents the first systematic review of methods used to conduct DCTs, examining both quantitative and qualitative outcomes including facilitators, barriers, advantages, disadvantages, patient experience, and stakeholder perspectives.
- Lee JJ, Burbury K, Underhill C, et al. Exploring Australian regional cancer patients’ experiences of clinical trial participation via telehealth. *Journal of telemedicine and telecare*. 2022;28:508-16.
  - ○ This reference is of importance as it describes a mixed methods analysis exploring the acceptability of teletrials, one form of DCTs, in regional and remote patients with cancer. It finds that teletrials are well-accepted by patients and their carers.

## Data Availability

No datasets were generated or analysed during the current study.
